# Experimental assessment of the safety and potential efficacy of high irradiance photostimulation of brain tissues

**DOI:** 10.1038/srep43997

**Published:** 2017-03-09

**Authors:** Senova Suhan, Scisniak Ilona, Chiang Chih-Chieh, Doignon Isabelle, Palfi Stéphane, Chaillet Antoine, Martin Claire, Pain Frédéric

**Affiliations:** 1 Neurosurgery Department, Assistance Publique-Hôpitaux de Paris (APHP), Groupe Henri-Mondor Albert-Chenevier, PePsy department, Créteil, F-94000, France; 2 U955 INSERM IMRB eq.14 Université Paris 12 UPEC, Faculté de Médecine, F-94010 Créteil, France; 3 IMNC, CNRS Univ. Paris Sud, Univ. Paris Saclay Orsay F-91405, France; 4 Faculty of Physics, Univ. Warsaw, P-02-093 Poland; 5 Department of Biomedical Engineering and Environmental Sciences, National Tsing-Hua University, Hsinchu city, 300, Taiwan; 6 Laboratory of Cellular interactions and liver physiopathology, INSERM, Univ. Paris-Sud, Univ. Paris Saclay, Orsay, F-91405 France; 7 L2S, CentraleSupélec, Univ. Paris Saclay, Gif sur Yvette, F-91192 France; 8 Univ. Paris Diderot, Sorbonne Paris Cité, Unité de Biologie Fonctionnelle et Adaptative, CNRS F-75205, Paris, France

## Abstract

Optogenetics is widely used in fundamental neuroscience. Its potential clinical translation for brain neuromodulation requires a careful assessment of the safety and efficacy of repeated, sustained optical stimulation of large volumes of brain tissues. This study was performed in rats and not in non-human primates for ethical reasons. We studied the spatial distribution of light, potential damage, and non-physiological effects *in vivo,* in anesthetized rat brains, on large brain volumes, following repeated high irradiance photo-stimulation. We generated 2D irradiance and temperature increase surface maps based on recordings taken during optical stimulation using irradiance and temporal parameters representative of common optogenetics experiments. Irradiances of 100 to 600 mW/mm^2^ with 5 ms pulses at 20, 40, and 60 Hz were applied during 90 s. *In vivo* electrophysiological recordings and post-mortem histological analyses showed that high power light stimulation had no obvious phototoxic effects and did not trigger non-physiological functional activation. This study demonstrates the ability to illuminate cortical layers to a depth of several millimeters using pulsed red light without detrimental thermal damages.

In the last decade, optogenetics has become common in most basic research neurobiology laboratories. This versatile technique has made it possible to explore brain networks due to the accurate activation or inhibition of specifically targeted cell populations^1^. At the same time, the use of brain stimulation or neuromodulation to cure or alleviate symptoms of severe diseases has gained interest for a wide scope of clinical applications. This approach was initially proposed and successfully applied to relieve the tremors due to Parkinson’s disease, and is currently under investigation for the treatment of several major diseases including obsessive-compulsive disorders, severe depression, Alzheimer disease, epilepsy or addiction^2^,^3^,^4^,^5^,^6^. Clinical neuromodulation relies on electrical stimulation through permanently implanted electrodes. Among alternatives, such as repeated transcranial magnetic^7^ or ultrasound stimulation^8^, optical control of genetically engineered cells (optogenetics), presents several advantages: the selectivity of stimulated cells, the high spatio-temporal resolution of neuronal control, the compatibility of optical fibers with MRI, and minimally invasive implementation when illumination is achieved using optical fibers positioned at the brain surface. Optogenetics studies on animal models may also help to refine the design of DBS protocols^6^.

The translation of optogenetics to non-human primates (NHP) models and its eventual clinical application^9^,^10^,^11^ still faces two major challenges. First, it requires an efficient and safe gene transfer technique to incorporate exogenous DNA in humans. Issues, such as long term expression, the effect of overexpression, and immunogenicity must be carefully addressed before clinical application. Several approaches are currently under investigation in clinical trials to address these issues, which are critical, but outside the scope of the present study^12^,^13^,^14^,^15^,^16^,^17^. The second challenge is the triggering of light sensitive opsins located in deep cortical layers, several millimeters beneath the brain surface, as epidural photostimulation would be less invasive. Opsins that can be triggered by red-light readily address this technical challenge as only the red and infrared wavelengths, in the so-called optical clinical window, exhibit low tissue absorption and moderate scattering. Initially only sensitive to blue light, red-shifted opsins are now available, potentially allowing non-invasive activation or inhibition of deep cortical layers^18^. However, the optical irradiance required to activate or inhibit light sensitive channels within the tissues is assumed to be between 1 and 5 mW/mm^2^ for most opsins^19^,^20^. To obtain this level of irradiance at a depth of 3 mm, corresponding to the thickness of the NHP cortex, would theoretically require much more power (in the range of 200–500 mW/mm^2^) at the surface, depending on the targeted cerebral area and the blood content of the tissues. Such powers levels are likely to generate thermal effects, of which their evaluation is a key milestone on the translational path of optogenetics.

The purpose of the present study was to investigate the safety and efficacy of *in vivo* brain sustained stimulation with red light with irradiances and frequencies relevant for optogenetics neuromodulation over large brain volumes. This is a key issue for the clinical translational community before chronic optical modulation of brain cells using optogenetics can be considered and evaluated in NHP models. We evaluated the potential occurrence of damage or non-physiological effects due to illumination and resulting heat with the perspective of applications to NHP and, eventually, patients. For comparative purposes, we also conducted the study using blue light, at 476 nm, the preferred wavelength for the triggering of the wild-type rhodopsin channel (ChR2), widely used in neuroscience research.

We performed our study in rats for ethical reasons despite the higher anatomo-functional behavioral similarities between NHP and with humans^17^. We exposed the brains of anesthetized rats to increasing epidural red light irradiance and increasing stimulation frequencies. The use of lateral optical stimulation allowed us to record axial surface maps. These data were compared to those from similar experiments using blue light. For both experimental conditions, we obtained 2D spatial maps of light distribution and thermal changes, as well as dynamic maps of thermal changes. Post-mortem studies were also conducted to assess the influence of physiological effects, such as blood flow, on thermal changes. Finally, histology and electrophysiology were carried out to investigate potential photo-toxicity or non-physiological brain activation due to optical stimulation.

## Results

### *In vivo* light distribution in the brain cortex

**Figure 1:**
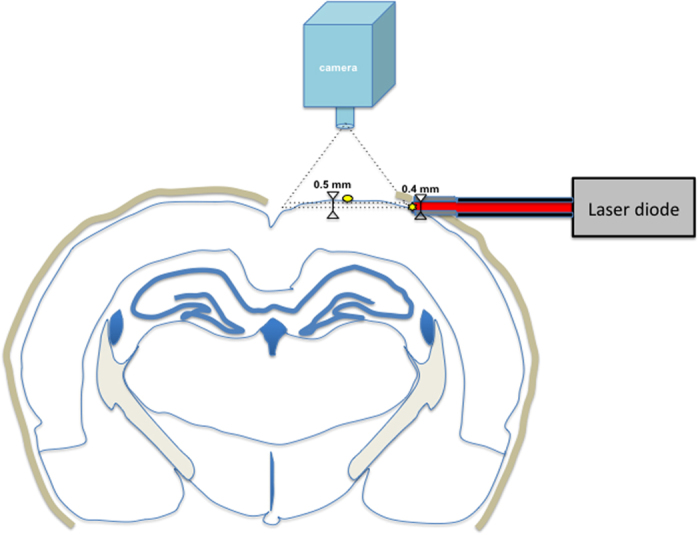
Scheme of a coronal slice of a rat brain showing the position of dorsal and lateral craniotomies. Dorsal craniotomy is centered at AP = −3.4 mm, ML = 2 mm (yellow dot) and is 4 × 7 mm^2^. Lateral craniotomy is centered at AP = −3.4 mm, ML = 4 mm, DV −0.7 mm (yellow star). The fiber core has a 0.4 mm diameter and is inserted through the lateral craniotomy and pressed in contact to the brain, so that there is no air gap in between. The thick brown line represents the skull.

**Figure 2:**
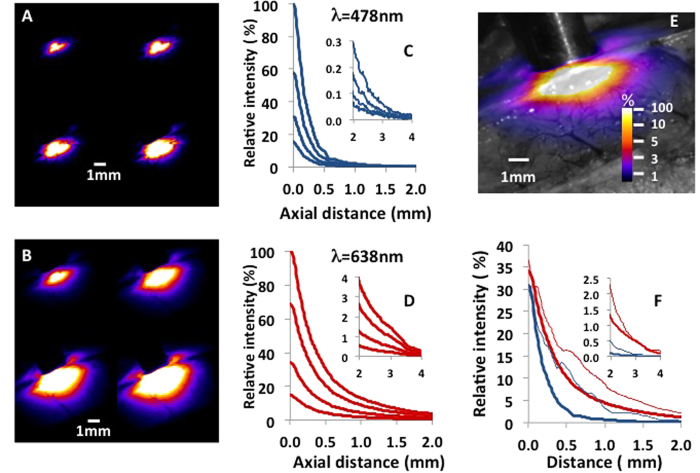
Relative spatial distribution of light for blue and red photostimulations. (**A**) Relative light distribution maps for blue light (λ = 476 nm) for increasing irradiance of photostimulation. From left to right and from top to bottom, 100 mW/mm^2^, 200 mW/mm^2^, 400 mw/mm^2^, and 600 mw/mm^2^. The color scale is shown in (**E**). (**B**) Relative light distribution maps for red light (λ = 638 nm). (**C** and **D**) Axial profiles of light distributions in (**A** and **B**). Data, from the bottom to the top curves, are profiles for 100 mW/mm^2^, 200 mW/mm^2^, 400 mw/mm^2^, and 600 mW/mm^2^ stimulation normalized against data recorded at 600 mW/mm^2^. Inserts: close-up for distances between 2 and 4 mm. (**E**) Overlay of relative light distribution map (200 mW/mm^2^λ = 638 nm) on an anatomical image of an exposed rat cortex. (**F**) Comparison of light distribution for blue and red stimulation at 200 mW/mm^2^. Thick solid lines are the axial profiles. Thin lines are the lateral profiles. Inserts: zoom for distances between 2 and 4 mm.

We first mapped the *in vivo* light distribution in the brain cortex to measure the relative light distribution of red and blue light at the surface of the brain tissues (Figs 1 and 2). Most of the blue light did not penetrate more than 500 μm below the surface (Fig. 2A), whereas the red light penetrated much deeper into the tissue (Fig. 2B) due to lower absorption and scattering, demonstrating the advantage of using red light for optogenetic stimulation. Increasing the irradiance level resulted in an increase of the illuminated volume for both wavelengths. The distribution maps also show the local inhomogeneity that is attributable to strongly absorbing and scattering blood vessels. In optogenetics using ChR2, the most currently used opsin, 1–5 mW/mm^2^ is assumed to be the minimum irradiance to systematically trigger cellular events^19^,^20^. The axial profiles (Fig. 2C and D) show that a 1 mW/mm^2^ threshold was obtained 1.1, 1.4, 1.7, and 2 mm below the surface for blue light irradiance from 100 up to 600 mW/mm^2^, whereas the corresponding depths for red light at the same irradiances was 2.8, 3.3, 3.7, and 4 mm. Using a more conservative threshold of 5 mW/mm^2^, the depths accessible to optogenetic stimulation in red light were reduced to 1.5, 2.1, 2.8 and 3.1 mm. A comparison of light distribution profiles for blue and red light (Fig. 2F) confirms that red light would be more efficient than blue light for opsin excitation deeper than 200 micrometers below the fiber tip. Surprisingly, light penetration perpendicular to the fiber axis was higher than that along the fiber axis for both wavelengths.

### The spatial extent of thermal effects is larger than the extent of light distribution extent but temperature increase remains low

**Figure 3:**
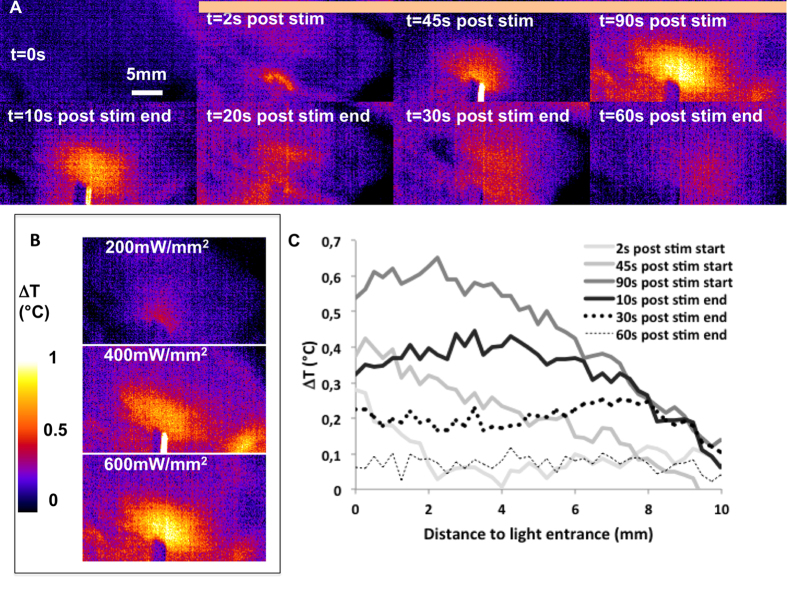
Spatial extent of thermal effect. (**A**) Spatio-temporal maps of thermal changes (representative single trial, stimulation parameters: 638 nm, 600 mW/mm^2^, 40 Hz for 90 s). The orange horizontal bar indicates the stimulation period. (**B**) Spatial extent of thermal effects at the end of 90 s photostimulations at 200, 400, and 600 mW/mm^2^, 40 Hz, 638 nm (average of 5 trials for each condition). (**C**) Axial profile of thermal changes at times corresponding to individual maps in (**A**).

We generated thermal maps (Fig. 3), similar to the light distribution maps. The thermal maps showed thermal effects that were wider on the lateral axis than on the axial axis of the fiber, similar to that of light distribution, but displayed a much larger spatial extent. However, it is not strictly possible to compare the spatial extent of irradiance and thermal effects since the recorded signals result from the combination of different biophysical and instrumental parameters. 2D thermal maps were recorded at the surface of the cortex, and represent thermal changes integrated on about 100 μm of tissue in the depth axis. Furthermore, heat is continuously and rapidly transported in the brain tissues due to diffusion and convection mechanisms.

The temperature rapidly increased following the onset of light stimulation (Fig. 3A). The temperature of the brain tissues increased by approximately 0.3 °C, close to the fiber, less than 2 s after the onset of the light pulses of intense red light stimulation (600 mW/mm^2^ at 40 Hz). Effects were measurable 1 mm away from the fiber tip. By 45 s, the temperature increase extended to approximately 6 mm from the fiber tip, but remained moderate (0.4 °C) even for this high irradiance stimulation. At the end of the stimulation, the temperature increase was measurable in tissue located 8–10 mm from the fiber tip. The temperature changes remained below 1 °C, even for this extremely intense and sustained stimulation. The temperature decreased rapidly after the end of the light stimulation, returning back to baseline after approximately 60 s (more rapidly for less intense stimulation). Both the temperature increase and spatial extent were lower when we applied lower power stimulation (Fig. 3B). A 200 mW/mm^2^ stimulation led to a maximum increase of less than 0.3 °C, confined to within a few millimeters of the fiber tip. The axial profiles of temperature changes corresponding to each time point from Fig. 3A are shown on Fig. 3C. At 2 and 45 s after the onset of stimulation, the spatial profile of temperature increase showed a progressive decrease from the fiber tip. At the end of the stimulation period, we observed a flat profile up to 3–4 mm from the fiber, indicating that the temperature was homogeneous over a large area. After the end of the stimulation, the temperature decreased homogenously returning to baseline showing a flat axial profile. For example, the remaining heat was distributed throughout the tissue 30 s after the end of light stimulation (Fig. 3A). For each tested condition, the temperature changes induced by blue and red light were not statistically significant for any tested condition (p > 0.05).

### Temporal temperature changes are moderate and show highly reproducible patterns

**Figure 4:**
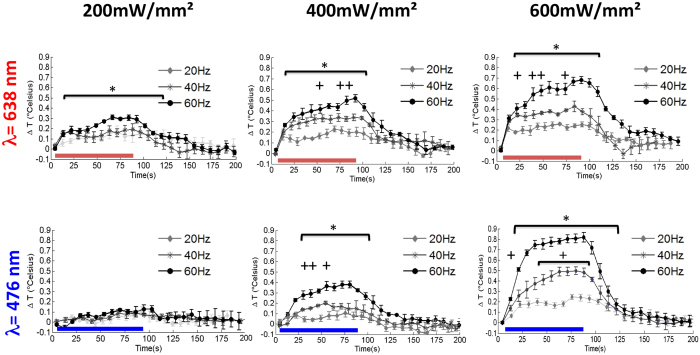
Time courses of temperature changes as a function of optical power densities and stimulus frequencies. (n = 5, data are represented as the mean +/− s.e.m). Data were derived from a 1 mm^2^ region of interest drawn around the hottest spot, close to the fiber tip. Horizontal bars indicate when the optical stimulation is on. Data have been binned in 8 s temporal windows for the sake of clarity. Stars and crosses indicate statistical significance for non-parametric Wilcoxon signed-rank tests for comparisons between data obtained at 60 and 20 Hz and data obtained at 40 and 20 Hz, respectively (p < 0.05).

We drew a circular region of interest (ROI) with an area of 1 mm^2^ around the hottest point for the analysis of the 2D temperature change maps. We calculated the mean value of pixels inside this ROI for each time point to derive the time course of temperature changes for single trials, and plotted the average time courses across all trials acquired using the same stimulation parameters for both red- and blue-light stimulation (Fig. 4). The temporal pattern was highly reproducible for identical stimulation parameters. The maximum temperature increased with frequency for a given irradiance (e.g. from 0.2 °C at 20 Hz to 0.8 °C at 60 Hz for 600 mW/mm^2^ at 638 nm). The time-courses followed a similar pattern for all experimental conditions. The temperature first increased steeply within seconds following the onset of stimulation onset (see also Fig. 3A). There was then an inflexion in the slope of the temperature increase approximately 10 to 20 s after the onset of stimulation. The temperature reached a plateau after the inflexion, before the stimulation ended (e.g. 400 mW/mm^2^, 40 Hz) except for the most intense stimulation parameters, where the temperature continued to increase at a uniform rate until the end of stimulation (400 mW/mm^2^ at 60 Hz and 600 mW/mm^2^ at 40 and 60 Hz). We observed a small and consistently reproducible decrease for some temporal profiles corresponding to moderate irradiance after the initial temperature increase (e.g. small temperature drops at 35 s for 400 mw/mm^2^, 20 Hz, or at 60 s for 600 mw/mm^2^ 20 Hz), suggesting thermal feedback control from the tissue. After the end of optical stimulation, the temperature decreased with a time course symmetrical to that observed during the rapid increase phase. The temperature returned to baseline within 90 s after the end of the stimulation for all tested conditions. The results of the 2D maps reported here reflect the changes in the tissues located 100 μm or less from the brain surface. This important point is further discussed in the discussion section.

### Temperature increases linearly with optical stimulation

**Figure 5:**
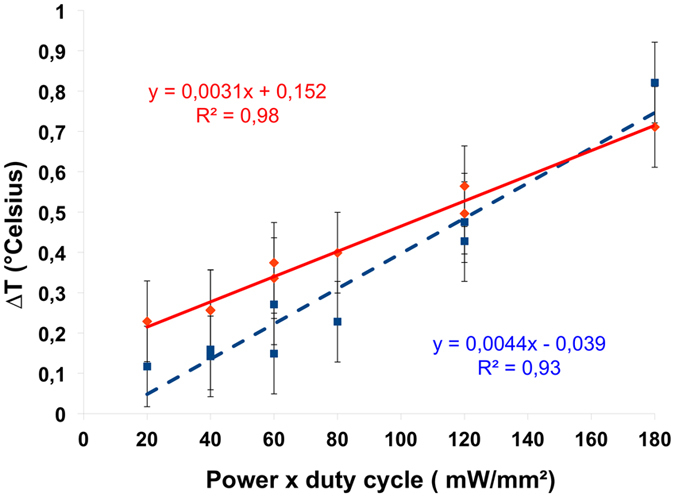
Linear relationship between maximal temperature increase and power x duty cycle used as stimulation parameters. The maximal temperature corresponds to the averaged maximum temperature increase from data of Fig. 3. The solid line shows the linear fit for the data obtained for red illumination, and the dashed line for blue illumination.

We studied whether the temperature increase is lower when using lower frequency pulses with accordingly adjusted irradiance to provide the same total amount of energy. There was a linear relationship between the maximal temperature increase and the product of the power and duty-cycle of the stimulation (Fig. 5). This demonstrates that the heat distribution is not affected by the frequency for the equivalent irradiance in the range suited for optogenetics (20–60 Hz). As a consequence, temperature changes are similar for different stimulation parameters provided that the product of irradiance and the duty cycle is the same. For example, a power x duty cycle of 120 corresponds either to 400 mW/mm^2^, 60 Hz or 600 mW/mm^2^, 40 Hz. The linear regression of these data allows prediction of the maximum temperature increase at the fiber tip for a given irradiance and frequency.

### Assessment of temperature induced effects at the cellular level

**Figure 6:**
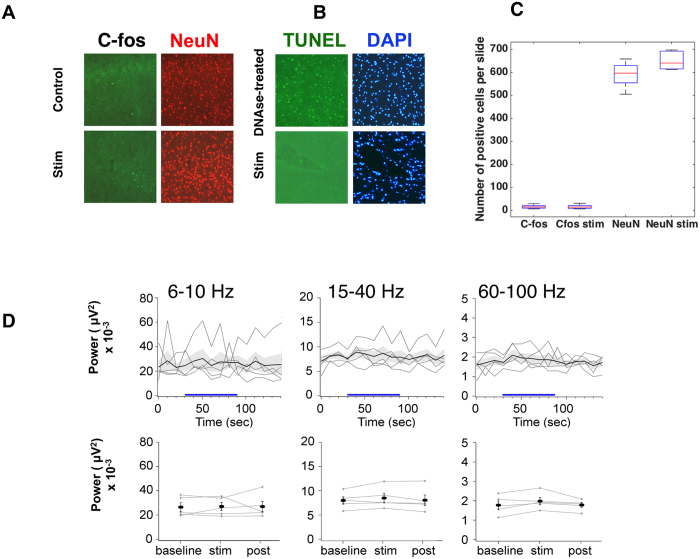
Optical stimulation does not produce brain activation or cellular toxicity. Histology. (**A** and **B**) Representative slides of NeuN, C-fos, TUNEL, and DAPI stained control and photostimulated sides from the same animal; (**C**) Box and whisker plots for C-fos and NeuN staining for stimulated and control sides. (**D**) Local field potentials do not change during optical stimulation. The local field potential recorded in the cortical area illuminated by the fiber did not change during photostimulation (blue line). Top row: LFP power processed by time bins and averaged across recording sessions performed at five different electrode positions (light trace) and mean value (bold trace) +/− s.e.m (shaded area). Bottom row. LFP power averaged for baseline, stim, and post periods, for the five electrode positions (light trace) and mean value (bold bar) +/− s.e.m.

We performed immuno-histology after blue- or red-light simulation at 200 mW/m2 and 40 Hz. Representative data for NeuN, C-fos, DAPI, and TUNEL immunostaining after red light stimulation are shown in Fig. 6A–C. Immunostaining of the neuron cellular body (NeuN) shows no differences in the number of cells between light-stimulated and the contralateral control cortex, indicating that sustained optical stimulation does not induce short-term gross damage, resulting in cell loss. We also examined c-fos immunostaining, an early marker of neuron activation, in the same brain regions to search for unwanted neuronal activation, independent of optogenetic mechanisms. As expected, the density of c-fos immunoreactive cells was low and was not significantly modified by sustained optical stimulation. Finally, brain tissues exposed to repeated sustained photostimulation did not show staining with TUNEL, a marker of DNA fragmentation resulting from apoptosis (Fig. 6B).

We confirmed these results by recording electrophysiological local field potential (LFP). We repeatedly performed population recordings for five electrode positions, (7 trials per position) during a sequence of red optical stimulation, measuring individual and mean LFP power across time in the α (6–10 Hz), β (15–40 Hz) and γ (60–100 Hz) frequency bands (Fig. 6D). We observed no significant changes of LFP power for either individual traces or the corresponding average for any of the frequency bands during the simulation phase relative to baseline and recovery phases (Friedman test between the 3 phases, n = 5 trials, p = 0.14, 0.13, 0.14, respectively).

## Discussion

### Limitations of analytical and Monte Carlo approaches to light penetration and thermal effects in optogenetics

Analytical calculations and Monte Carlo (MC) simulations have been used for decades in the field of biomedical optics to evaluate irradiance distribution. However, they are generally considered to be a first intention tool for comparing or optimizing instrumentation design or experimental protocols. Their predictive power concerning quantification presents severe limitations. Analytical calculations in the field of optogenetics have used the Kubelka Munk approach^21^,^22^, which relies on assumptions, such as isotropic scattering or isotropic illumination, that are inconsistent with real-life optogenetics experiments as discussed recently^23^. Regarding MC simulations, strong limitations also arise from the enormous simulation times associated with complex voxelized heterogeneous geometries^24^ and lack of validated optical properties measured *in vivo*, despite on-going efforts^25^,^26^. In this context, our experimental light distribution measurements reveal that the extent of light distribution relative to the fiber is larger in the lateral than axial axis, which is not reported in the vast literature of analytical calculations or Monte Carlo simulations of fiber guided light in cerebral tissue^21^,^23^,^27^. Although the fiber output is an extended source and not a point source, this result was unexpected, considering the high forward diffusion anisotropy of brain tissue. A likely explanation is that MC simulations of light propagation, in a single homogeneous material with optical properties affected by a large degree of uncertainty, are not able to capture complex light conduction mechanisms at the metal ferule/brain/dura-matter/skull interfaces. As an example, comparison of the measured light distribution profiles at 476 and 638 nm with those numerically estimated previously^21^ reveals that the experimental data show a large underestimation of red light penetration, due to an oversimplified geometry, inaccurate optical transport calculations, and optical tissue properties. Efforts are ongoing to improve the speed and accuracy of MC simulations^24^,^28^, some specifically dedicated to optogenetics^23^,^29^,^30^, but the substantial effect of the uncertainty of brain optical parameters on simulation output still prevent them from quantitatively predicting either light distribution or thermal effects. Furthermore, the physiology of thermal regulation in the brain involves several mechanisms and structures including skull, cerebrospinal fluid, scalp, and cerebral blood flow (as shown from the comparison of *in vivo* and *ex vivo* temperature increase dynamics in Supplementary Figure 3) and may be affected by experimental protocols and methodology, which are difficult to take into account in simulations^31^. Consequently, we believe simulations should be considered with caution, when designing brain optogenetic experiments, and involve at least layered structure geometry for the skull, dura matter, and cerebrospinal fluid. The resulting light and heat distributions should be validated experimentally whenever possible.

### Temperature increases during typical optogenetic stimulation paradigms depends highly on the stimulation parameters

The superficial temperature increase was below 1 °C, on average, for all tested simulation parameters. We repeatedly found similar temperature changes for identical stimulation protocols for both wavelengths. The general agreement that “tissue absorbs more blue light than red light” should lead to higher temperature changes under blue excitation. Closer examination of the data from the literature shows that the absorption and diffusion coefficients for brain tissues are slightly but significantly lower at 638 nm than at 476 nm^24^,^25^, the two wavelengths used in our study. Blue light penetrated less the brain tissue than red light as shown on Fig. 2, but, the irradiances rapidly homogenized and decreased with increasing distance, due to the high optical diffusion of the brain tissues at both wavelenghts. The significant temperature increases for the wavelengths used in our study were localized within the first few hundred microns around the fiber tip. In addition, the rapid heat diffusion in tissues are likely to mask the limited differences arising from the different absorption of blue and red light.

Considering the relation between optical absorption and temperature changes, a simple calculation based on optical energy absorption and heat capacity should lead to a much larger temperature increase^22^. This suggests that simple calculations based on the heat capacity of water are not accurate. The use of more relevant models, such as the well-validated Bioheat equation^32^, as implemented recently^30^, are more appropriate. Furthermore, we found a strong linear relationship between the maximum temperature recorded at the fiber tip and the product of the continuous irradiance and the duty cycle. This demonstrates the presence of additive thermal effects when the stimulation pulse frequency is increased from 20 to 60 Hz or the irradiance from 200 to 600 mW. This heat “piling-up” effect has been previously observed under blue illumination (445 nm, 20% or 50% duty cycle, irradiance from 78 to 1032 mW/mm^2^)^33^ and infrared stimulation for pulse frequencies above 5 Hz^34^. Further work needs to be performed to verify whether similar additive effects occur for different pulse durations (1–10 ms). However, the pulse duration evaluated here is typical of optogenetic experiments, and shorter pulses would not be adapted to typical opsin kinetics^1^. Guided by a translational perspective, we did not perform experiments using continuous illumination. However, if prolonged continuous stimulation were required, the choice of step function opsins would be more appropriate since they only need to be stimulated by a brief pulse of light at the beginning and end of the desired period of illumination^35^.

### Methodological issues and comparison with previous thermal measurements during optical stimulation

The temperature increase observed in our experiments was significantly smaller than previous experimental findings in which temperature increases of up to several °C were reported during optical stimulation^30^,^33^. The 2D maps in our study reflect the temperature changes measured at the surface of the brain. This point is important for the interpretation of the results. We illuminated the brain through a craniotomy made over the temporal cortex using a 400 μm diameter fiber. The optical axis was set 500 μm below the brain surface. Temperature changes are monitored at the surface of the frontal cortex (Fig. 1). We used this strategy to obtain an estimate of the temperature profile in the coronal plane when the brain is illuminated using a light source applied anywhere in the dorsal aspect, corresponding to the most common experimental configurations. We have made Monte Carlo simulations of the photon transport in brain tissues to evaluate the influence of total reflection at the brain-air interface on the irradiance and thermal effect maps (Supplementary Figure 2). The simulation showed that less that 2% of all the photons launched from the optical fiber into the brain tissues, undergone total reflection at the brain-air interface. Moreover, we compared the temperature increase recorded by the thermal imager, as well as a miniature thermocouple implanted into the tissue close to the light entry point and from 0 to 1 mm below the brain surface. This experiment showed that the maximal temperature increase in the superficial maps was about 40% lower than the temperature increase in the immediate vicinity of the fiber tip (Supplementary Figure 1). This suggests that a corrective factor of approximately 1.5 should be applied to thermal imager data to determine the highest temperature increase, resulting in a temperature increase ranging from 0.15 °C to 2.5 °C for single trials under our experimental conditions. Brain temperature increases up to 7.5 Celsius degrees due to photostimulation with irradiances several orders of magnitude higher than those used for optogenetics were successfully detected with out set-up (Supplementary Figure 4). The difference between our results and those from previous studies are thus not discrepancies but mostly reflect the influence of several experimental parameters: animal preparation, total irradiance, fiber diameter, and stimulation pattern (wavelength, total duration, pulse width and frequency).

Here, we derived irradiance (in mW/mm^2^) by measuring the power at the output of the fiber using continuous (not pulsed) illumination. This power is then divided by the cross section of the fiber. This is the standard method used by commercial providers of optogenetic hardware who offer lasers and LED sources in the range of 50 mW/mm^2^ to 1000 mW/mm^2^. In the recent study of Stujenske *et al*., Monte Carlo simulations and experimental validation were carried out for 532 nm optical stimulation during 60 s with 10 or 20 mW at the output of a 62 micron diameter fiber^30^. This led to temperature increases of 3 to 6 °C, but these irradiances correspond to 3312 and 6624 mW/mm^2^. These irradiances, coupled with the use of 532 nm light (where the absorption coefficient of brain tissues peaks at a local maximum due to the contribution of hemoglobin) and the lower numerical aperture (0.22 vs 0.48) could explain the difference observed in the vicinity of the fiber between our study and that of Stujenske *et al*. It is important to underline that these differences tend to disappear with increasing distance from the fiber tip because of homogenization of the irradiance due to optical diffusion in the first millimeter from the fiber tip (see Supplementary Figure 6 in ref. 30).

In another study, a 5 °C increase was observed. However, this was after stimulation at 445 nm of 1,062 mW/mm^2^ (continuous) pulsed using a high (50%) duty cycle with very long pulses (20 ms)^33^. The parameters used in the present study (476 or 638 nm, 100–600 mW/mm^2^, duty cycle 10–30%, 5 ms pulses) are more representative of those commonly used for optogenetics experiments in rodents. These comparisons show the importance of controlling the optical fiber geometrical characteristics (numerical aperture and diameter) and optical stimulation paradigms. A larger diameter or a lower duty cycle for the same optical power led to lower irradiance and minimized thermal effects.

### Temperature changes are within the physiological range

Many studies have been conducted on the influence of brain temperature on cellular and tissue physiological viability because of its clinical importance in post-stroke treatment or photodynamic therapy. Several reviews^31^,^36^,^37^ provide an overview of the mechanisms involved at the cellular, tissues and blood compartment level. Physiological fluctuations of brain temperature related to sleep, arousal, sensory stimulation, and environmental challenges have been reported in many animal models including monkeys^38^,^39^, and rodents^40^,^41^,^42^,^43^,^44^. In these studies, the range of local temperature increase was 0.1 °C–2.5 °C in anesthetized animals, which was enhanced in awake and freely moving animals. Using realistic optical stimulation parameters for deep brain optogenetic stimulation, we observed an average maximal temperature increase of approximately 0.9 °C at the brain surface and 1.5 °C inside the tissues. These changes are within the range of changes reported under normal physiological conditions.

Red light irradiance of 100–400 mW/mm^2^ at the surface is required to reach the opsin triggering threshold several millimeters below the brain surface. The thermal effects following irradiation using standard stimulation parameters (up to 30% duty cycle during 90 s) did not produce visible tissue alterations. TUNEL staining of frontal slices did not show increased DNA degradation at any distance from the fiber tip (Fig. 6B). This apparent absence of cellular damage is in agreement with cellular studies that showed only rare and reversible photothermal effects (deactivation of enzymes) for transient temperature increases of up to 43 °C^36^. Studies in the infrared range have reported thermal damage in brain tissues for radiant exposure above 0.4 J/cm^2^. Here, the tissue optical absorption coefficients for the visible blue and red light stimulation of the brain are dominated by hemoglobin absorption and are approximately 100-fold lower, implying that tissues can withstand much higher radiant exposure before tissue damage occur.

### No evidence of light induced changes in LFP in the brains of wild type mice during or after optical stimulations

We found no evidence of neuronal activation at the population level, either in the expression of the immediate early gene C-fos (Fig. 6A) or in the characteristics of the local field potential *in vivo* (Fig. 6D). Physiological effects were previously observed, either as increased neuron firing rates^30^ or changes in blood oxygen level dependent (BOLD) functional magnetic resonance (fMRI) signals^33^. Stujenske *et al*. showed neuronal activity, presumably evoked by a temperature increase due to green light stimulation (532 nm). Sustained illumination increased the firing rate of neurons located 500 μm from the light delivering optical fiber tip. As already discussed, the local irradiances used in their study were several orders of magnitude higher than those commonly applied in optogenetic experiments and used in our study. Blue light-induced heating of the cerebral cortex of wild type mice has also been shown to cause profound positive and negative fMRI responses depending on the irradiance and duty cycle^33^. The effect on BOLD fMRI signals is mild for irradiances leading to a 1 °C increase, but become stronger when the temperature increase reaches 2 °C and above. This effect is attributed to changes in temperature driven relaxation times. *Ex vivo* measurements showed the same artefacts, ruling out a contribution of physiological changes. Our post-mortem studies support these findings. The maximal temperature reached at the end of the stimulation was similar *in vivo* and *ex vivo*, despite the presence of physiological mechanisms *in vivo* that transiently diminish temperature changes (Supplementary Figure 3). Concerning the interpretation of artefactual fMRI signals, it is likely that the effect of temperature driven changes of relaxation times are much greater than slight and transient blood flow changes. Neuronal activation under infrared light stimulation has been widely studied^34^,^45^,^46^. Radiant exposure of as little as 0.01 J/cm^2^ of wavelengths near 1470 and 1900 nm, corresponding to water absorption peaks, activates neurons^34^,^45^,^47^. The photo-thermal origins of such neuronal activation remain incompletely understood^48^,^49^. The optical absorption coefficients are dominated by hemoglobin absorption for the optical illumination of brain tissue at 476 and 638 nm and are approximately 10- to 100-fold lower than in the infrared, suggesting that the threshold for neuronal activation was not reached in our stimulation paradigms.

## Conclusion

This study demonstrates that repeated and sustained photostimulation of deep cortical brain tissues, performed using irradiances in the range of those delivered by commercially available fiber-optic-based laser sources at 638 nm, is minimally deleterious for brain tissue and function. There was no short term thermal damage, either at the tissue or cellular level, nor light-induced activation of neuronal populations in the cerebral cortex. Our experimental data also highlight that simulations or analytical calculations of light and heat distribution within brain tissues following illumination should be performed with care, due to their simplified geometries and the lack of validated experimental values on optical and thermal properties of brain tissues. Piling up of the heat effect occurs within the range of tested parameters (irradiance 100 mw/mm^2^−600 mW/mm^2^, 10–30% duty cycle, 5 ms pulses) resulting in a linear relationship between maximum temperature and the product of the irradiance and the duty cycle. This provides guidelines for the design of optical stimulation protocols.

## Experimental procedures

### Animal preparation

All experiments were performed in accordance with European Directive 86/609/EEC, concerning the care and use of laboratory animals. The experimental protocol was examined and approved by the French veterinary authorities (DDPP authorization B91471101). Six rats (Wistar) were used in this study with a mean weight of 250+/−50 g. Stereotaxic surgery was performed under anesthesia by intraperitoneal injection (0.3 ml/50 g) of a cocktail composed of medetomidine (0.5 mg/kg), and ketamine (75 mg/kg). The level of anesthesia was confirmed by toe pinch and absence of ocular reflex. Body temperature was monitored and maintained at 37 °C throughout the experiment, using a heating blanket. The temperature of the room was kept constant. Two craniotomies were performed under stereotactic surgery. A sagittal craniotomy (approximately 1 × 1 mm^2^, centered on AP: −3.4 mm, ML = 4 mm, DV = −0.7 mm, relative to the bregma) was performed to allow the positioning a single optical fiber orthogonal to the left temporal cortex surface, at its most dorsal coordinate. An axial craniotomy (approximately 4 × 7 mm^2^, centered on AP = −3.4 mm, ML = 2 mm, relative to the bregma) was performed to expose the surface of the left frontal cortex and allow recording of the spatial distribution of the light and temperature changes during light stimulation to generate the spatiotemporal maps. The detailed configuration of the craniotomies and fiber position is shown in Fig. 1.

### Light stimulation

Epidural light stimulation was carried out using either a 638 nm red laser (Doric Lenses Inc., Canada) or a 476 nm blue laser (Shanghai Laser, China). The term “power” is confusing and often misleading in the literature, so we use the physical terms of irradiance, which is the light power flux per unit of surface. Both lasers were operated under fibered conditions, using a 400 μm diameter glass fiber with a numerical aperture of 0.48. The fiber tip was positioned orthogonal to the tissue with gentle pressure to avoid air gaps between the fiber tip and the tissue. Light irradiance at the fiber tip was verified using a power-meter (PM100 with S130C detector, Thorlabs USA) for continuous illumination. Continuous irradiance was applied at 100, 200, 400, 600, mW/mm^2^. External modulation was successively set to 20, 40, and 60 Hz. The width of the light pulses was 5 ms. The corresponding duty cycles and average power are listed in Supplementary Table 1. A 10-s background period was recorded before the 90-s stimulation was started. Acquisitions were successively repeated five times for all imaging experiments described below to determine experimental variability. The inter-stimulus interval was at least 3 min.

### Optical imaging set–up

The optical imaging set-up consisted of a stereomicroscope (MZ16, Leica) associated with a fast CMOS camera (Orca Flash 2.0, Hamamatsu). Images of light diffusion into the brain tissues were acquired for continuous illumination at 100, 200, and 600 mW/mm^2^ using the same exposure time (20 ms) for the sake of comparison. Reflectance images showing the vasculature were obtained using wide field illumination in green (530 nm) light to check that no bleeding occurred following cranial window surgery.

### Thermal imaging set-up

The thermal imaging setup consisted of a thermal camera (Gobi 384, Xenics, USA) with a sensitivity of 0.08 °C. It was used to record movies (3 frames per s) of the exposed rat brain before (10 s), during (90 s) and after (120–150 s) optical stimulation. The thermal images result from the integration of temperature changes over the first hundred microns of the brain surface. To avoid bias in the temperature change measurements, due to ambient temperature changes, the experiments were carried out in an experimental room heated to 25 °C. The absolute measurements at the brain surface were in the 28–31 °C range, which is obviously below the body temperature but is very similar to what was reported in the neocortex of animals undergoing craniotomies for brain imaging studies^50^. The thermal imager was calibrated for each experiment by simultaneously recording the cooling of a beaker filled with hot water using a fast thermocouple, (Pico Technology, UK) and the thermal camera. Raw grey level images from the thermal camera were calibrated and scaled using ImageJ to obtain spatio-temporal maps of thermal changes (in °C) relative to baseline. To measure the temperature elevation at the fiber tip inside the rodent brain, a miniature thermocouple (type T, 0.01 °C sensitivity, 0.05 mm diameter, Omega, UK) was stereotaxically implanted. The thermal imager was simultaneously used to acquire thermal changes at the brain surface. This experiment showed that the temperature increase at the fiber tip was approximately 40% higher than that recorded at the brain surface by the thermal imager (Supplementary Figure 2).

### Histology

Two animals (one for each exposure wavelength) were used for histology. They were exposed to repeated (5 trials, 5 min inter-stimulus interval) epidural light stimulation, at 200 mW/mm^2^ and 40 Hz for 90 s, applied at the surface of the left temporal cortex as described above. The rats were injected with a lethal dose of pentobarbital and decapitated 30 min after end of the last stimulation. The brain was removed and immediately frozen at −80 °C. Serial coronal brain sections (10 μm thickness) were cut using a cryostat (Leica) for both the ipsilateral and contralateral (self-control) hemispheres of the brain with respect to light stimulation. All sections were fixed with 4% para-formaldehyde. Some sections were permeabilized with Triton-X (0.2%) and labeled using the TUNEL cell death detection kit (Roche Diagnostics). Positive controls were treated with DNAse (Qiagen) prior to labeling. Other sections were incubated 48 h at 4 °C in primary antibodies: anti-C-fos 1:500 (Santa Cruz) and anti-Neu-N 1:250 (Millipore). The sections were then incubated in Alexa-conjugated secondary antibodies. Quantification and image analysis were conducted using a Zeiss-AxioLab-A1 fluorescence Microscope. A 16x objective was used to count the number of positive C-fos nuclei and the number of positive Neu-N neurons in 12 consecutive sections of the region of interest and the same region of the contralateral hemisphere.

### Electrophysiology

Electrophysiological activity was recorded during light stimulation. The dura was removed and a microelectrode (microTargeting^TM^, FHC, ME; impedance 0.8–1.5 MΩ at 1 kHz) inserted in five distinct recording sites, located 200 μm in the latero-medial axis from the spot of light penetration in the brain, and 150 μm in the antero-posterior axis. The tip of the electrode was lowered to 300 μm. Neural data were amplified (x1000; P511 AC amplifier Grass Technologies, West Warwick, RI, USA), filtered (0.3–10 000 Hz), digitized at 20 kHz using a CED Power1401 converter (Cambridge Electronic Design, Cambridge, UK), and stored for off-line analysis. The signal was recorded for 30 s before photostimulation (baseline), 90 s during photostimulation at 200 mW/mm^2^ and 40 Hz (stim), and 120 s after the end of photostimulation (post). The same protocol was repeated five times for each position of the electrode. Data were processed using OpenElectrophy software (http://packages.python.org/OpenElectrophy^51^). A continuous Morlet wavelet transform was applied to frequency bands of interest resulting in an estimate of the oscillatory power for each time and frequency value. We obtained time frequency power maps, where each level of energy is represented for each time and frequency value. Power was extracted for time windows of 3 s of signal every 10 s. Analyses were conducted separately for three frequency bands: 6–10 Hz; 15–40 Hz, and 60–100 Hz. Statistical analyses were performed using nonparametric Friedman tests to compare power values between the three conditions: baseline, stim, and post.

## Additional Information

**How to cite this article**: Suhan, S. *et al*. Experimental assessment of the safety and potential efficacy of high irradiance photostimulation of brain tissues. *Sci. Rep.* **7**, 43997; doi: 10.1038/srep43997 (2017).

**Publisher's note:** Springer Nature remains neutral with regard to jurisdictional claims in published maps and institutional affiliations.

## Supplementary Material

Supplementary Figures and Table
