# Erratum: Experimental assessment of the safety and potential efficacy of high irradiance photostimulation of brain tissues

**DOI:** 10.1038/srep46199

**Published:** 2017-04-07

**Authors:** Suhan Senova, Ilona Scisniak, Chih-Chieh Chiang, Isabelle Doignon, Stéphane Palfi, Antoine Chaillet, Claire Martin, Frédéric Pain

Scientific Reports 7: Article number: 43997; 10.1038/srep43997 published online 03 09 2017; updated: 04 07 2017.

The original version of this Article contained errors in the spelling of the authors Suhan Senova, Ilona Scisniak, Chih-Chieh Chiang, Isabelle Doignon, Stéphane Palfi, Antoine Chaillet, Claire Martin & Frédéric Pain which were incorrectly given as Senova Suhan, Scisniak Ilona, Chiang Chih-Chieh, Doignon Isabelle, Palfi Stéphane, Chaillet Antoine, Martin Claire & Pain Frédéric respectively.

These errors have now been corrected in the HTML and PDF versions of this Article.

