# Supplementary material for: Experimental assessment of the safety and potential efficacy of high irradiance photostimulation of brain tissues
Source: Sci Rep. 2017 Mar 9;7:43997. doi: 10.1038/srep43997 (PMC5343659; doi:10.1038/srep43997)
Supplement: Supplementary Figures and Table [file srep43997-s1.pdf]

Experimental assessment of the safety and potential efficacy of high irradiance photostimulation of brain tissues

Senova Suhan[1,2], Scisniak Ilona[3,4], Chiang Chih-Chieh[3,5], Doignon Isabelle[6], Palfi Stéphane[1,2], Chaillet Antoine[7], Martin Claire[3,8], Pain Frédéric[3*]

Supplementary Figure 1. Comparison of thermal camera and thermocouple measurements. A/ Experimental set-up. The thermocouple is mounted on a XYZ micrometric arm to control the depth of its sensitive tip inside the tissues. B/ Temperature rise measured by the thermocouple at the immediate vicinity of the fiber tip for increasing total average power. The area filled with grey dots corresponds to the regime used for measurements with the thermal camera. C/ Temperature rise as a function of depth (85mW total average power). The thermocouple is positioned in the plane perpendicular to the optical axis of the fiber. D/ Relationship between the temperature measured at the fiber tip inside the tissue (thermocouple measurement) and the maximal temperature rise measured at the surface with the thermal camera.

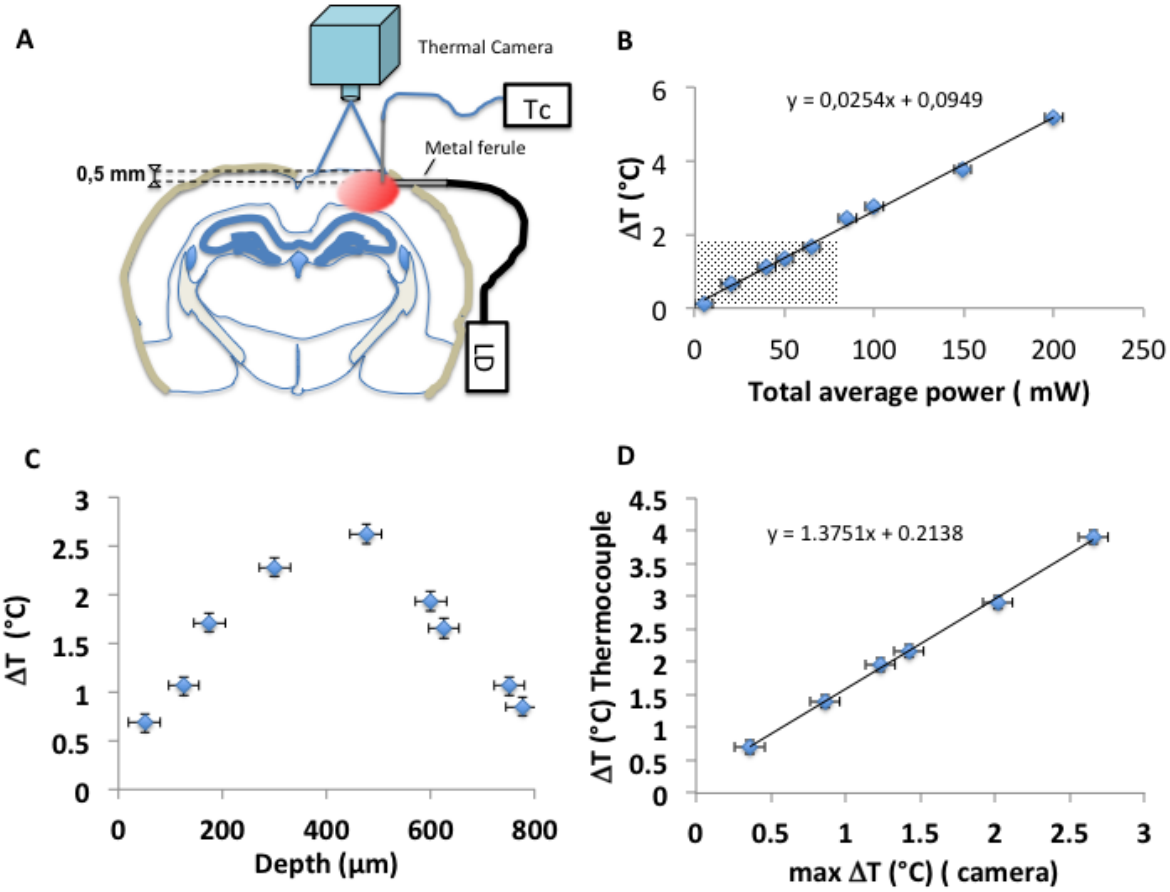

Supplementary Figure 2. Monte Carlo simulations of the photon tracks in the brain tissue ruled out a potential bias of irradiance or heat maps due to total reflection at the brain/air interface. A) Geometry of the Monte Carlo simulation B) Optical parameters used in the optical simulation C) The results of the Monte Carlo simulation show that less than 3% of the photons that enter the brain undergo a total reflection at the brain/air interface.

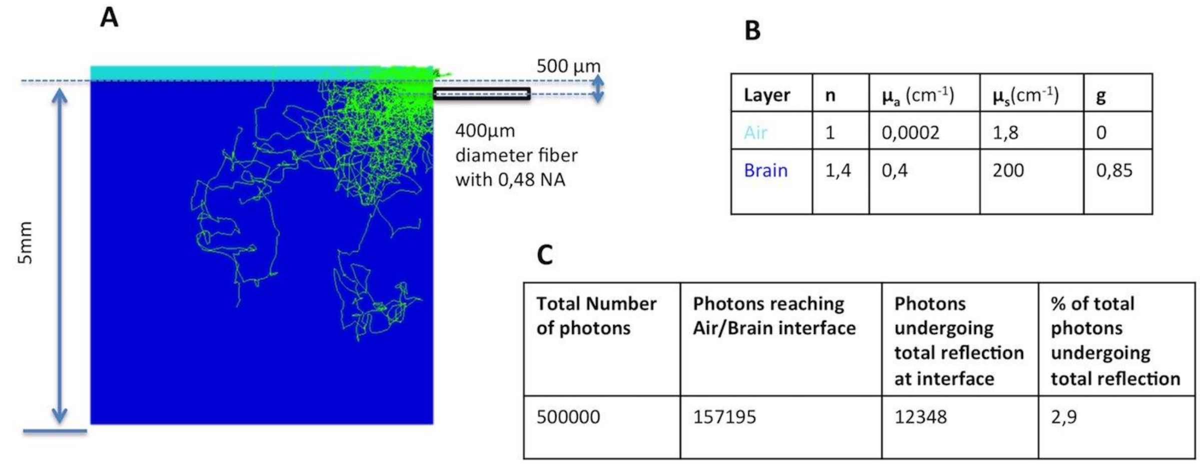

B

Layer	n	μ_a (cm^{-1})	μ_s(cm^{-1})	g
Air	1	0,0002	1,8	0
Brain	1,4	0,4	200	0,85

C

Total Number of photons	Photons reaching Air/Brain interface	Photons undergoing total reflection at interface	% of total photons undergoing total reflection
500000	157195	12348	2,9

Supplementary Figure 3: Post-mortem versus *in vivo* temperature changes. Stimulation was performed with red light at 200 mW/mm², 40 Hz, data averaged on five consecutive trials, represented as mean +/- s.e.m). Red line, black diamonds correspond to post-mortem measurements. Black line, grey squares correspond to in vivo measurements. The red horizontal bar indicates when the optical stimulation is on. For clarity, data have been binned in 8 s temporal windows. Stars indicate statistical significance ($p<0.01$) for non-parametric Wilcoxon signed-rank tests.

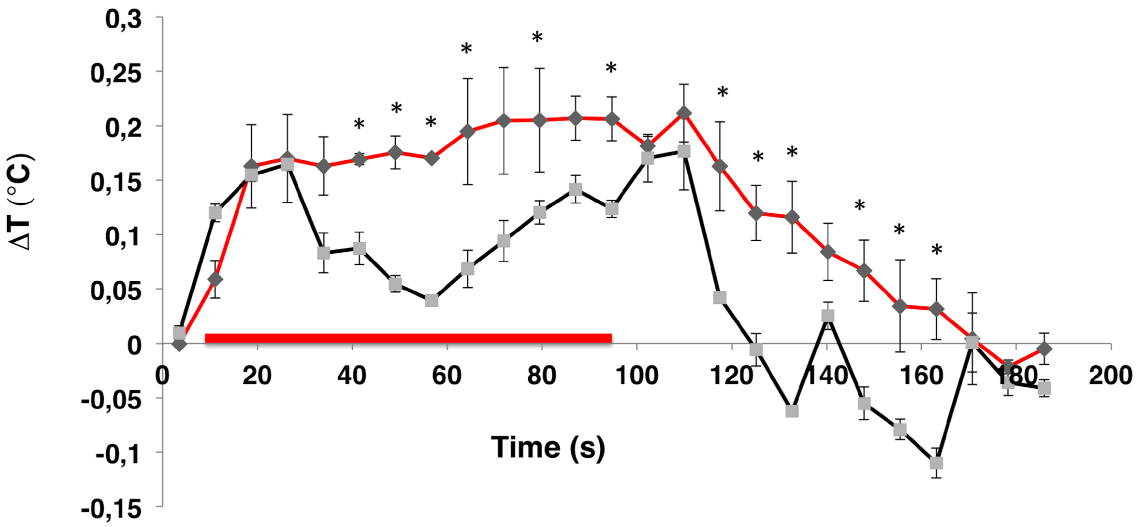

Supplementary Figure 4: Temperature changes for very high continuous irradiances

Optical stimulations were performed *in vivo* in one animal with red light during 75s (red bar) using an equivalent continuous illumination of 3000mW/mm^2 (grey graph) and 6000mW/mm^2 (black graph) leading to maximum temperature increases of 3.9 and 7,5°C respectively. This allowed to assess the ability of the experimental set-up to detect very high surface temperature increases.

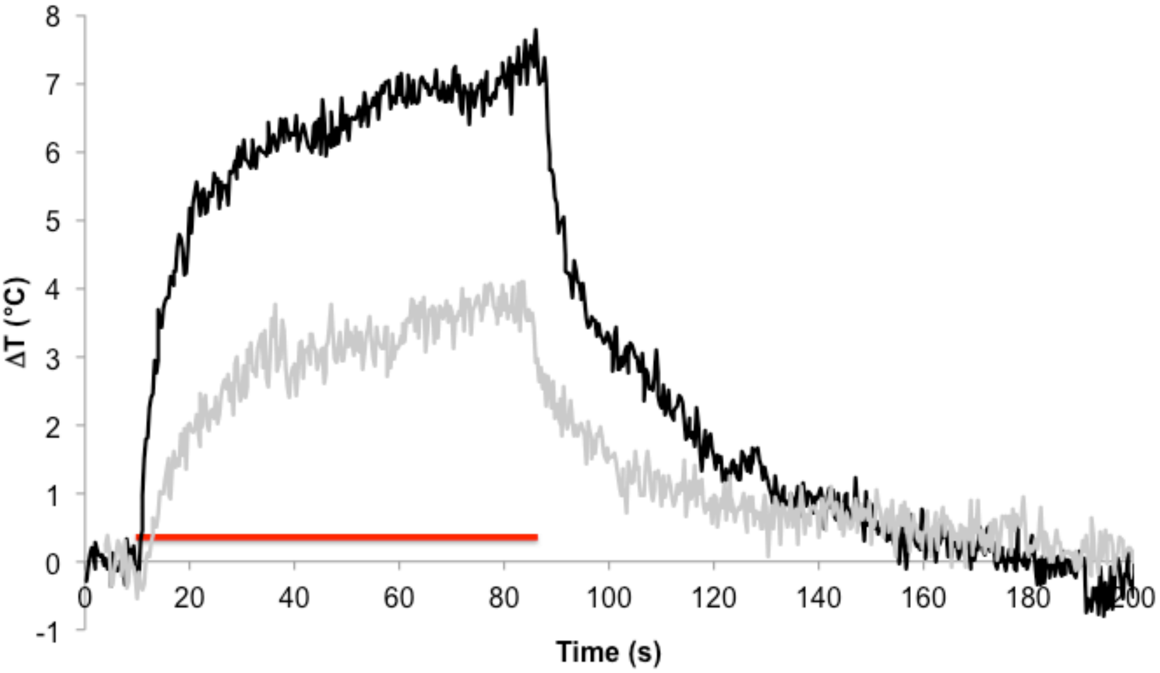

Supplementary Table 1. Average continuous power delivered for the different stimulation conditions used in the study.

Irradiance mW/mm2	Continuous power (mW)	Average power (5ms, 20hHz) Duty-cyle 10%	Average power (5ms, 40Hz) Duty-cyle 20%	Averaged power (5ms, 60Hz) Duty-cyle 30%
100	12,6	1,3	2,5	3,8
200	25,1	2,5	5,0	7,5
400	50,3	5,0	10,1	15,1
600	75,4	7,5	15,1	22,6
